# Cardiac Sarcoidosis: A Practical Guide for Cardiologists

**DOI:** 10.1007/s11886-026-02417-w

**Published:** 2026-09-30

**Authors:** Massimo Imazio, Francesco Venturelli, Valentino Collini

**Affiliations:** Department of Medicine, University of Udine, University Hospital Santa Maria della Misericordia, ASUFC, Udine, University of Udine, Piazzale Santa Maria della Misericordia 15, Udine, 33100 Italy

**Keywords:** Cardiac sarcoidosis, Diagnosis, Multimodality imaging, Therapy, Prognosis

## Abstract

**Purpose of Review:**

Cardiac sarcoidosis (CS) is an inflammatory granulomatous cardiomyopathy associated with atrioventricular block, ventricular arrhythmias, heart failure (HF), and sudden cardiac death (SCD). Diagnosis and management remain challenging because myocardial involvement is patchy and evidence from randomized trials is limited. This review summarizes current knowledge on CS, focusing on advances in diagnosis, immunosuppression, and prevention of arrhythmic events.

**Recent Findings:**

Cardiac involvement is clinically recognized in 5–10% of patients with sarcoidosis but is more frequent in imaging and autopsy studies. Cardiac magnetic resonance (CMR) and ^18F-fluorodeoxyglucose positron emission tomography (FDG-PET) provide complementary information on myocardial scar and active inflammation. Multimodality imaging, extracardiac tissue diagnosis, guided endomyocardial biopsy, and consensus criteria improve diagnostic confidence. Corticosteroids remain first-line therapy for active inflammation, while steroid-sparing agents are increasingly used to reduce corticosteroid exposure or treat persistent disease. Inflammasome/interleukin-1 inhibition is an emerging targeted strategy, but its clinical efficacy remains unproven. SCD risk stratification now extends beyond left ventricular ejection fraction to include ventricular arrhythmias, conduction disease, ventricular dysfunction, and myocardial scar burden. Implantable cardioverter-defibrillator therapy remains central in selected high-risk patients.

**Summary:**

CS requires early recognition of both inflammation and myocardial scar. CMR and FDG-PET are central to diagnosis, prognosis, and therapeutic decisions, while management combines immunosuppression, HF therapy, and arrhythmia prevention. Major uncertainties remain regarding optimal diagnostic criteria, immunosuppression strategies, serial imaging, and primary-prevention ICD selection. Prospective studies and randomized trials are needed to refine treatment and risk stratification.

## Epidemiology

Sarcoidosis is a systemic granulomatous disease with an estimated prevalence of approximately 35 per 100,000 individuals in the United States, with higher incidence rates observed among African American populations [1, 2]. Clinically evident cardiac sarcoidosis (CS) is identified in about 5–10% of patients with systemic sarcoidosis; however, autopsy studies suggest myocardial involvement in 20–30% of cases, indicating that many cases remain undiagnosed during life [3, 4].

In Japan, CS is diagnosed more frequently and accounts for a higher proportion of sarcoidosis-related deaths, with some studies reporting cardiac involvement in up to 60% of autopsy cases [5]. Conversely, registry data from the United States estimate that cardiac involvement contributes to 10–25% of sarcoidosis-related deaths [3].

CS typically affects adults between the ages of 40 and 60 [6]. Advancements in imaging modalities, such as cardiac magnetic resonance imaging (MRI) and positron emission tomography (PET), along with increased clinical awareness, have led to improved detection and earlier diagnosis of CS in recent decades [7].

## Etiopathogenesis

The precise cause of sarcoidosis remains elusive. It is hypothesized that an environmental or infectious antigen triggers an exaggerated cell-mediated immune response in genetically predisposed individuals. Certain human leukocyte antigen (HLA) alleles, particularly HLA-DRB1 variants, have been associated with increased susceptibility and severity of sarcoidosis across different ethnic groups [8].

Proposed environmental and infectious antigens include mycobacterial and propionibacterial peptides, mold, insecticides, and silica exposure; however, none have been definitively established as causative agents. Notably, *Propionibacterium acnes* has been detected within sarcoid granulomas, suggesting a potential etiological link [9].

In sarcoidosis, activated CD4⁺ T-helper type 1 (Th1) lymphocytes release interleukin-2 (IL-2), interferon-gamma (IFN-γ), and tumor necrosis factor-alpha (TNF-α), promoting macrophage activation and the formation of noncaseating granulomas, which are the pathological hallmark of the disease [10]. These granulomas commonly affect the basal interventricular septum and left ventricular free wall, particularly in subepicardial regions [11]. Over time, the inflammatory phase, characterized by edema and active granulomatous inflammation, may transition to a chronic fibrotic phase. Importantly, granulomas often exhibit a patchy and heterogeneous distribution within affected tissues [12].

### Clinical Presentation

Cardiac sarcoidosis (CS) presents a broad clinical spectrum, ranging from asymptomatic disease to sudden cardiac death (SCD). Subclinical cardiac involvement is identified in approximately 20–30% of cases, often detected only through advanced imaging or routine screening in patients with systemic sarcoidosis [13, 14]. However, clinically manifest CS may present dramatically, and several “red flag” scenarios should prompt suspicion.

#### **Conduction Disturbances**

Unexplained high-grade atrioventricular (AV) block in a patient under 60 years old is a classic presentation. Complete heart block is the most frequent conduction abnormality in CS, occurring in approximately 23–30% of cases, and up to 44% in those with histologically confirmed disease [13]. Bundle branch block—especially right bundle branch block or left anterior fascicular block—and axis deviations on ECG are also suggestive in the appropriate clinical context [13].

#### **Ventricular Arrhythmias**

**Fig. 1:**
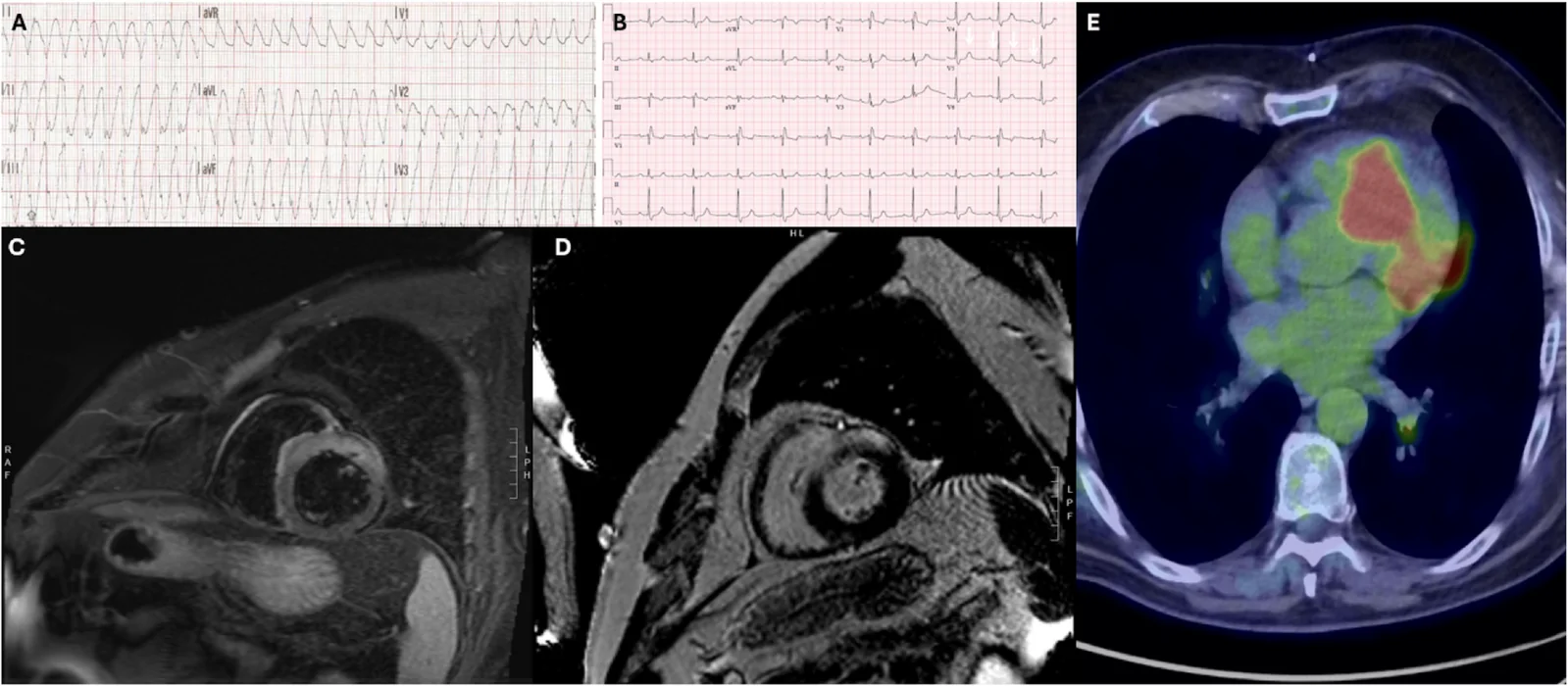
Arrhythmic Presentation of Cardiac Sarcoidosis in a 62-Year-Old Patient. Panel **A**: Electrocardiogram (ECG) demonstrating ventricular flutter. Panel **B**: Baseline ECG showing sinus rhythm with atrioventricular block (AVB) and intraventricular conduction abnormalities, including right bundle branch block and left anterior fascicular block. Panels **C** and **D**: Cardiac magnetic resonance imaging (MRI) using TIRM (C) and late gadolinium enhancement (LGE) (D) sequences, revealing a prominent area of myocardial edema and delayed contrast uptake in the anterior septum and anterior walls of both the left and right ventricles. This pattern is consistent with the characteristic “hook sign” of cardiac sarcoidosis. Panel **E**: Fluorodeoxyglucose positron emission tomography (FDG-PET) showing intense radiotracer uptake in the same regions, correlating with MRI findings and indicating active myocardial inflammation

Frequent premature ventricular contractions (PVCs) or non-sustained ventricular tachycardia (VT) on ambulatory monitoring may be early indicators of CS (Fig. 1). Cohort studies report that 30–32% of patients with CS develop VT or frequent PVCs [15]. Notably, life-threatening arrhythmias can occur even when left ventricular ejection fraction (LVEF) is preserved. Males appear to have a higher incidence of these arrhythmias [15].

#### **Heart Failure**

Some patients present with progressive LV systolic dysfunction and symptoms of heart failure (HF). However, isolated HF at initial presentation is relatively uncommon, accounting for < 20% of cases [3, 4, 16]. In the early inflammatory phase, diastolic dysfunction may predominate due to granulomatous infiltration and myocardial edema, while the later fibrotic phase is associated with reduced systolic function [3, 4].

#### **Other Features**

Less commonly, CS presents with chest pain caused by coronary vasculitis or microvascular dysfunction, pericardial tamponade, or valvular abnormalities. Ventricular aneurysms—especially in the basal septum or anterior wall—are observed in ~ 5–10% of CS cases and typically reflect localized scarring in more advanced disease [17].

Up to 25% of CS cases occur in the absence of known extracardiac sarcoidosis. These isolated cases lack systemic symptoms and can be particularly difficult to diagnose. In such cases, the first clinical manifestation is often a cardiac event such as high-grade AV block or VT [18].

#### **Risk of Sudden Cardiac Death**

SCD is a leading cause of mortality in CS, accounting for 25–65% of disease-related deaths [19]. Ventricular tachyarrhythmias (VT/VF) are the most common cause, though complete heart block can also lead to SCD. Identified risk markers for SCD include prior sustained VT or cardiac arrest, unexplained syncope, LVEF ≤ 35%, NYHA class III–IV symptoms, extensive late gadolinium enhancement (LGE) on cardiac magnetic resonance imaging (CMR), and inducible arrhythmias on electrophysiologic testing [19]. Importantly, these predictors are not always reliable—some patients with normal LVEF or minimal symptoms still experience malignant arrhythmias [3, 4, 19].

In summary, CS should be considered in otherwise healthy individuals—particularly younger adults—presenting with unexplained AV block, arrhythmias, or cardiomyopathy. Its presentation may mimic idiopathic arrhythmias or non-ischemic dilated cardiomyopathy, often leading to misdiagnosis.

### Diagnostic Testing

Diagnosing cardiac sarcoidosis (CS) is inherently complex, as no single test offers definitive sensitivity or specificity. Consequently, a multimodal diagnostic approach is recommended, integrating clinical evaluation with various imaging and laboratory assessments.

#### Electrocardiogram (ECG)

ECG serves as a fundamental screening tool, revealing abnormalities in approximately 30–50% of CS cases. Findings may include atrioventricular blocks, bundle branch blocks, axis deviations, Q waves, or ventricular ectopy. However, a normal ECG does not exclude CS [3, 4].

#### Laboratory Tests

While no blood test is specific for CS, certain markers can suggest disease activity. Elevated serum angiotensin-converting enzyme (ACE) levels may be observed in sarcoidosis patients, though their sensitivity and specificity are limited [20]. Similarly, elevated troponin or B-type natriuretic peptide (BNP) levels can indicate myocardial involvement but are non-specific.

#### Echocardiography

Transthoracic echocardiography (TTE) is a first-line imaging modality for CS, capable of detecting chamber enlargement, regional wall motion abnormalities, aneurysms, or basal septal thinning. Reduced left ventricular ejection fraction (LVEF) may be evident in advanced stages. An abnormal TTE in a sarcoidosis patient warrants further evaluation with cardiac magnetic resonance imaging (CMR) or positron emission tomography (PET) [3, 4].

#### Cardiac Magnetic Resonance (CMR)

CMR is pivotal in diagnosing and risk stratifying CS. Late gadolinium enhancement (LGE) detects myocardial fibrosis or granulomatous inflammation with high sensitivity (~ 95%). Typical findings include patchy, non-ischemic LGE involving the mid-myocardium or subepicardium, especially in the basal septum and lateral walls. The extent of LGE correlates with prognosis; involvement of ≥ 5–6 segments or ≥ 10% of LV mass is associated with increased risk of ventricular arrhythmias and mortality [21].

#### FDG-PET Imaging

Fluorodeoxyglucose positron emission tomography (FDG-PET) complements CMR by identifying active inflammation. Active granulomas exhibit increased glucose uptake, while perfusion imaging reveals scarred or inflamed myocardium. Multifocal cardiac FDG uptake with extracardiac involvement has near-100% specificity for CS, with overall sensitivity around 80–90%. Serial PET scans can assess treatment response by showing decreased uptake [22].

#### Endomyocardial Biopsy (EMB)

Histologic evidence of noncaseating granulomas via EMB remains the diagnostic gold standard for CS. However, its sensitivity is limited (~ 20–30%) due to the patchy nature of the disease. Image-guided or electroanatomic-guided biopsies, targeting areas of active inflammation or scar identified by FDG-PET or CMR, can enhance diagnostic yield to approximately 50% [3, 4, 23].

#### Electrophysiology Study (EPS)

EPS aids in arrhythmic risk stratification, particularly in patients with LVEF > 35% but high-risk features on imaging. Programmed stimulation may reveal inducible ventricular tachycardia, supporting the decision for implantable cardioverter-defibrillator (ICD) placement. EPS can also localize conduction disease or guide ablation strategies. However, a negative EPS does not exclude future arrhythmic risk [24].

**Fig. 2:**
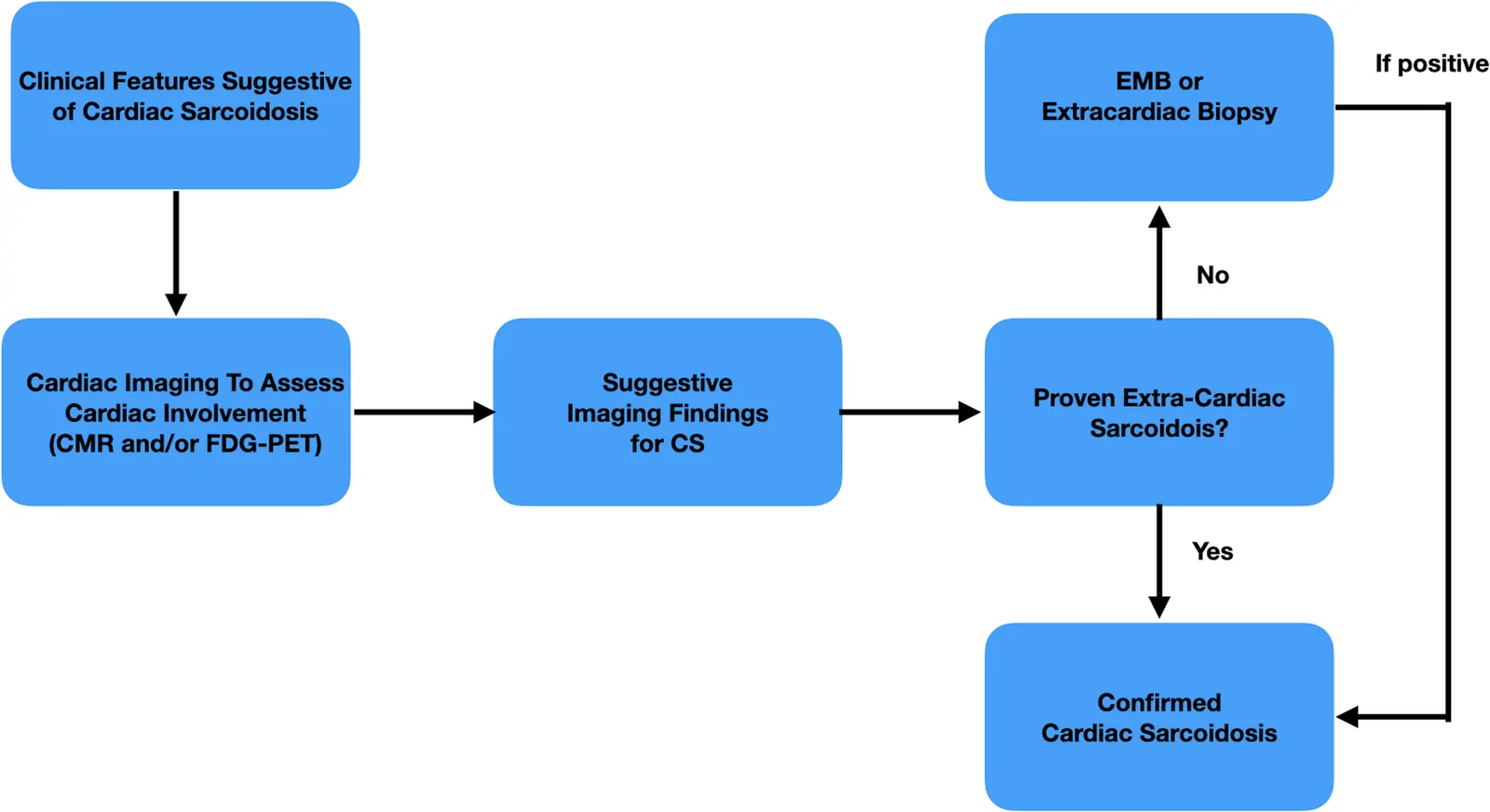
Diagnostic evaluation algorithm for suspected cardiac sarcoidosis. Abbreviations: AV, atrioventricular; CMR, cardiac magnetic resonance; CS, cardiac sarcoidosis; EMB, endomyocardial biopsy; FDG-PET, ^18F-fluorodeoxyglucose positron emission tomography; LBBB, left bundle branch block; LVEF, left ventricular ejection fraction; RBBB, right bundle branch block; VA, ventricular arrhythmia

A stepwise diagnostic approach typically begins with ECG, echocardiography, and CMR as initial assessments. If CMR reveals LGE suggestive of CS, FDG-PET is often performed to assess active inflammation and identify potential extracardiac sites for biopsy. A positive extracardiac biopsy combined with supportive cardiac imaging usually suffices for clinical diagnosis, obviating the need for EMB (Fig. 2).

### Diagnostic Criteria and Classification

Three major frameworks have been proposed to standardize the diagnosis of cardiac sarcoidosis: World Association of Sarcoidosis and Other Granulomatous Disorders Sarcoidosis Organ (WASOG) Assessment Instrument created in 1999 and updated in 2014 [25], the Heart Rhythm Society (HRS) international guideline document [26], and the Japanese Circulation Society (JCS) guidelines [27]. WASOG 2014, HRS 2014, and JCS 2016 diagnostic criteria are compared in Table 1. All incorporate clinical, imaging, and histologic elements, but they differ in their approach regarding the necessity of tissue confirmation and the recognition of isolated CS.

**Table 1:** Comparison of diagnostic criteria for cardiac sarcoidosis

Diagnostic Criteria	WASOG (2014) [25]	Heart Rhythm Society (HRS, 2014) [26]	Japanese Circulation Society (JCS, 2016) [27]
Histologic diagnosis	Non-caseating granulomas on biopsy, with exclusion of other causes	Non-caseating granulomas on biopsy, with exclusion of other causes	Non-caseating granulomas on biopsy, with exclusion of other causes
Clinical diagnosis pathway	Extracardiac biopsy-proven sarcoidosis AND one or more cardiac findings suggestive of CS: LVEF < 40% (unexplained); steroid-responsive cardiomyopathy or heart block; spontaneous or induced sVT in the absence of other risk factors; high-degree AV block; patchy FDG uptake on PET; LGE on CMR; positive gallium-67 scintigraphy; perfusion defect at scintigraphy or SPECT	Extracardiac biopsy-proven sarcoidosis AND one or more cardiac findings suggestive of CS: LVEF < 40% (unexplained); sustained VT; high-degree AV block; steroid-responsive cardiomyopathy or heart block; LGE on CMR; FDG uptake on PET; positive gallium-67 scintigraphy	A) Extracardiac histologic or strong clinical evidence of sarcoidosis. B) One of the following combinations: ≥2 major cardiac criteria; or 1 major + ≥ 2 minor cardiac criteria. Major criteria: reduced LVEF (< 50%); sustained VT or VF; advanced AV block; basal IVS thinning or aneurysm formation (LV/RV); patchy myocardial FDG uptake (PET); LGE in a non-ischaemic pattern (CMR); positive gallium-67 myocardial uptake. Minor criteria: frequent PVCs or non-sustained VT; bundle branch block or axis deviation; pathological Q waves on ECG; perfusion defects on nuclear imaging; abnormal myocardial texture on imaging; EMB showing fibrosis or monocyte infiltration without granulomas
Recognition of isolated CS	Not allowed	Not allowed	Allowed if clinical criteria are fulfilled
Exclusion of other causes	Required	Required	Required

*AV* atrioventricular, *CMR* cardiac magnetic resonance, *CS* cardiac sarcoidosis, *ECG* electrocardiogram, *EMB* endomyocardial biopsy, *FDG* ^18F-fluorodeoxyglucose, *HRS* Heart Rhythm Society, *IVS* interventricular septum, *JCS* Japanese Circulation Society, *LGE* late gadolinium enhancement, *LV* left ventricle, *LVEF* left ventricular ejection fraction, *PET* positron emission tomography, *PVC* premature ventricular contraction, *RV* right ventricle, *SPECT* single-photon emission computed tomography, *sVT* sustained ventricular tachycardia, *VF* ventricular fibrillation, *VT* ventricular tachycardia, *WASOG* World Association of Sarcoidosis and Other Granulomatous Disorders

#### **The WASOG criteria (2014)**

The WASOG criteria categorize CS likelihood into ‘definite,’ ‘probable,’ or ‘possible’ based on a combination of clinical presentation, imaging findings, and histological evidence. A ‘definite’ diagnosis of CS requires histological confirmation of non-caseating granulomas in myocardial tissue. WASOG criteria require histologic confirmation of sarcoidosis in at least one organ (not necessarily the heart) [25].

#### **The HRS consensus statement (2014)**

The HRS provides two diagnostic pathways: (1) histological confirmation of non-caseating granulomas in myocardial tissue, or (2) clinical diagnosis in patients with histologically confirmed extracardiac sarcoidosis plus one or more cardiac manifestations, including unexplained reduced LVEF (< 40%), sustained VT, high-degree AV block, or positive cardiac imaging findings. Similarly to WASOG, isolated CS without biopsy-proven disease remains outside the definitional scope of HRS.

#### **The JCS guidelines (2016)**

These guidelines differ fundamentally in allowing a diagnosis of CS even in the absence of any histologic confirmation. In addition to the histologic pathway (myocardial biopsy showing noncaseating granulomas), JCS permits a purely clinical diagnosis based on combinations of extracardiac and cardiac features. Notably, in certain scenarios, such as typical cardiac imaging findings in a patient with pulmonary or ocular sarcoidosis, a diagnosis can be made even without any tissue biopsy. This broader approach increases sensitivity, particularly for isolated CS, which JCS explicitly recognizes. Indeed, studies suggest the JCS criteria identify significantly more cases than WASOG or HRS, though with a potential trade-off in specificity.

In clinical practice, many centers adopt a pragmatic, hybrid approach seeking histologic confirmation of sarcoidosis from any organ, when feasible, and integrating cardiac imaging and clinical features to assess cardiac involvement. The 2024 American Heart Association (AHA) Scientific Statement endorses this integrative strategy, introducing a likelihood-based classification—definite, highly probable, probable, and possible—to guide clinical decision-making [3]. Similarly, the 2024 European Society of Cardiology (ESC) position paper reinforces a multimodal diagnostic approach, emphasizing the role of advanced imaging, clinical judgment, and timely immunosuppressive therapy for both isolated and systemic CS [4]. It also supports structured pathways that combine histologic, clinical, and imaging data to refine diagnostic accuracy and improve patient outcomes.

## Treatment

Management of CS is aimed at two primary objectives: (1) suppressing myocardial inflammation and (2) preventing and treating potentially life-threatening arrhythmias. This typically involves a combination of immunosuppressive therapy, device-based interventions for arrhythmias, and HF management when needed.

### **Immunosuppressive Therapy**

Immunosuppressive therapy remains the cornerstone of CS management, aiming to reduce inflammation, prevent arrhythmias, and improve cardiac function [28, 29].

Corticosteroids remain first-line treatment and are commonly initiated at 30–60 mg/day for 1–3 months, followed by a gradual taper over 6–12 months. Maintenance doses of 5–10 mg/day may be required to prevent relapse. Methotrexate (7.5–20 mg/week with folic acid supplementation) is the most widely used steroid-sparing agent. Azathioprine (1–2 mg/kg/day) and mycophenolate mofetil (1,000–1,500 mg twice daily) are alternatives when methotrexate is not tolerated or is contraindicated.

Evidence for azathioprine and mycophenolate mofetil is observational. In a 36-patient retrospective study, adding an immunosuppressant to corticosteroids (azathioprine in 5, methotrexate in 5, and cyclophosphamide in 2) was associated with fewer cardiac relapses than corticosteroids alone (16.7% vs. 45.8%), although agent-specific effects could not be determined [30]. In a 77-patient cohort, prednisone plus mycophenolate mofetil reduced cardiac FDG uptake and permitted lower corticosteroid exposure [31]. A 2025 electronic health record analysis of 601 patients with CS found no significant difference in serious cardiac outcomes among new users of methotrexate, mycophenolate mofetil, or azathioprine, but suggested a higher infection risk with mycophenolate mofetil and azathioprine than with methotrexate [32]. Thus, these agents are reasonable alternatives when methotrexate is unsuitable, but no randomized CS trial establishes their comparative efficacy.

Biologic therapy should be reserved for refractory active disease and selected within a multidisciplinary sarcoidosis program. Infliximab (3–5 mg/kg IV every 4–8 weeks) or adalimumab (40 mg SC every other week) may be considered after conventional steroid-sparing therapy fails. In a retrospective multicenter cohort of 38 patients (30 treated with infliximab and 8 with adalimumab), TNF-alpha inhibition reduced corticosteroid dose and improved or resolved cardiac FDG uptake in 73%, with stable mean LVEF; infections were frequent and four patients required HF hospitalization [33]. Rituximab is supported only by a seven-patient refractory CS series, in which FDG-PET inflammation decreased in six patients and LVEF improved or stabilized in four [34]. These uncontrolled data support consideration, not routine use, after standard therapy fails, with infection screening and monitoring and particular caution with TNF inhibitors in advanced HF. Suggested regimens are summarized in Table 2.

**Table 2:** Immunosuppressive Therapy for Cardiac Sarcoidosis

Therapy Line	Medication	Typical Dose	Duration	Clinical Notes
First-line	Prednisone	30–60 mg/day → 5–10 mg/day	1–3 mo, taper over 6–12 mo	Long-term use often needed
Second-line (associated with corticosteroids)	Methotrexate (MTX)	7.5–20 mg/week + folic acid	Long-term maintenance	Most used steroid-sparing agent
	Azathioprine	1–2 mg/kg/day	Long-term maintenance	Alternative when MTX is unsuitable; limited observational data; monitor infection [30, 32]
	Mycophenolate Mofetil	1000–1500 mg twice daily	Long-term maintenance	Retrospective steroid-sparing/PET data; monitor infection [31, 32]
Third-line (as alternatives in case of failure of second-line options)	Infliximab	3–5 mg/kg IV every 4–8 weeks	Based on response	Refractory active disease; screen TB/HBV; monitor infection and HF [33]
	Adalimumab	40 mg SC every other week	Based on response	Eight patients in the multicenter TNF-inhibitor cohort [33]
	Rituximab	1,000 mg IV once or twice, 2 weeks apart	Repeat individualized	Seven-patient refractory CS series; rescue use only [34]
Investigational / clinical trial	IL-1 blockade (anakinra; rilonacept)	Trial-specific	Pilot / ongoing phase II	Mechanistic/pilot evidence; not routine care [35–37]

Interest is also growing in the NLRP3 inflammasome/interleukin-1 (IL-1) axis as a mechanistic and therapeutic target. In surgical specimens from three patients with CS, inflammasome aggregates were present in all examined cardiac granulomas, providing mechanistic proof of concept [35]. The randomized, open-label MAGiC-ART pilot found that a 28-day course of anakinra was feasible and could modulate systemic inflammation, but it was not designed to establish clinical efficacy [36]. The ongoing randomized, open-label phase II REPAIR-CS trial is testing rilonacept added to standard nonbiologic therapy, with the 24-week change in myocardial inflammation on FDG-PET as its primary endpoint [37]. Until efficacy and safety are established, IL-1 blockade should be regarded as investigational and preferably used within clinical trials.

Monitoring involves clinical assessment and imaging, particularly FDG-PET. Cardiac FDG-PET is useful for assessing treatment response, with reduced uptake indicating remission. Guidelines recommend immunosuppressive treatment for all patients with definite or highly probable CS [3, 4]. Observation may be appropriate for asymptomatic patients with minimal or uncertain findings.

### **Heart Failure Management**

CS patients with systolic dysfunction or HF symptoms should receive standard guideline-directed medical therapy for HfrEF, including beta blockers, ACEi/ARBs (or ARNI), MRA, and SGLT2i, despite limited CS-specific trial data. Some may show EF recovery with timely immunosuppression. In cases of ventricular dyssynchrony with reduced EF, CRT may be beneficial, although evidence in CS is limited. For patients with advanced HF, referral for mechanical circulatory support or transplant should be considered [38, 39].

### **Arrhythmia Management and Device Therapy**

Cardiac sarcoidosis frequently necessitates a tailored approach to arrhythmia management due to its unique propensity for sudden cardiac death (SCD), often from ventricular tachycardia (VT), ventricular fibrillation (VF), or advanced atrioventricular (AV) block [3, 40, 41].

Current consensus guidelines assign a Class I recommendation for implantable cardioverter-defibrillators (ICDs) in CS patients with a history of cardiac arrest or sustained VT, and for primary prevention in those with a left ventricular ejection fraction (LVEF) ≤ 35% [3, 4]. However, because CS can present with preserved or mildly reduced EF, ICD implantation is also advised (Class IIa) for patients with LVEF between 36 and 49% who exhibit high-risk features—such as right ventricular (RV) involvement, unexplained syncope, late gadolinium enhancement (LGE) on cardiac magnetic resonance (CMR), or increased FDG uptake on positron emission tomography (PET) [3, 40, 41].

Patients requiring permanent pacing are generally recommended to receive an ICD instead of a standard pacemaker due to the elevated risk of ventricular arrhythmias [3, 40, 41]. Anti-arrhythmic drugs play a supportive role. Beta-blockers are commonly prescribed, especially when AV conduction remains unaffected. Amiodarone is often the agent of choice for recurrent VT and to reduce ICD shocks, though its long-term use may be limited by toxicity, and concerns of exacerbating pulmonary sarcoidosis. Pharmacologic therapy is considered adjunctive and does not replace the need for device-based protection [3, 40, 41].

Catheter ablation can be a valuable tool for reducing arrhythmic burden, particularly in cases of drug-refractory VT, that persist despite optimized immunosuppression and device therapy. The procedure targets scar-mediated re-entry circuits to suppress VT acutely. However, recurrence remains common, particularly if active myocardial inflammation persists or new fibrotic lesions form [42].

### Proposed Treatment Algorithm

**Fig. 3:**
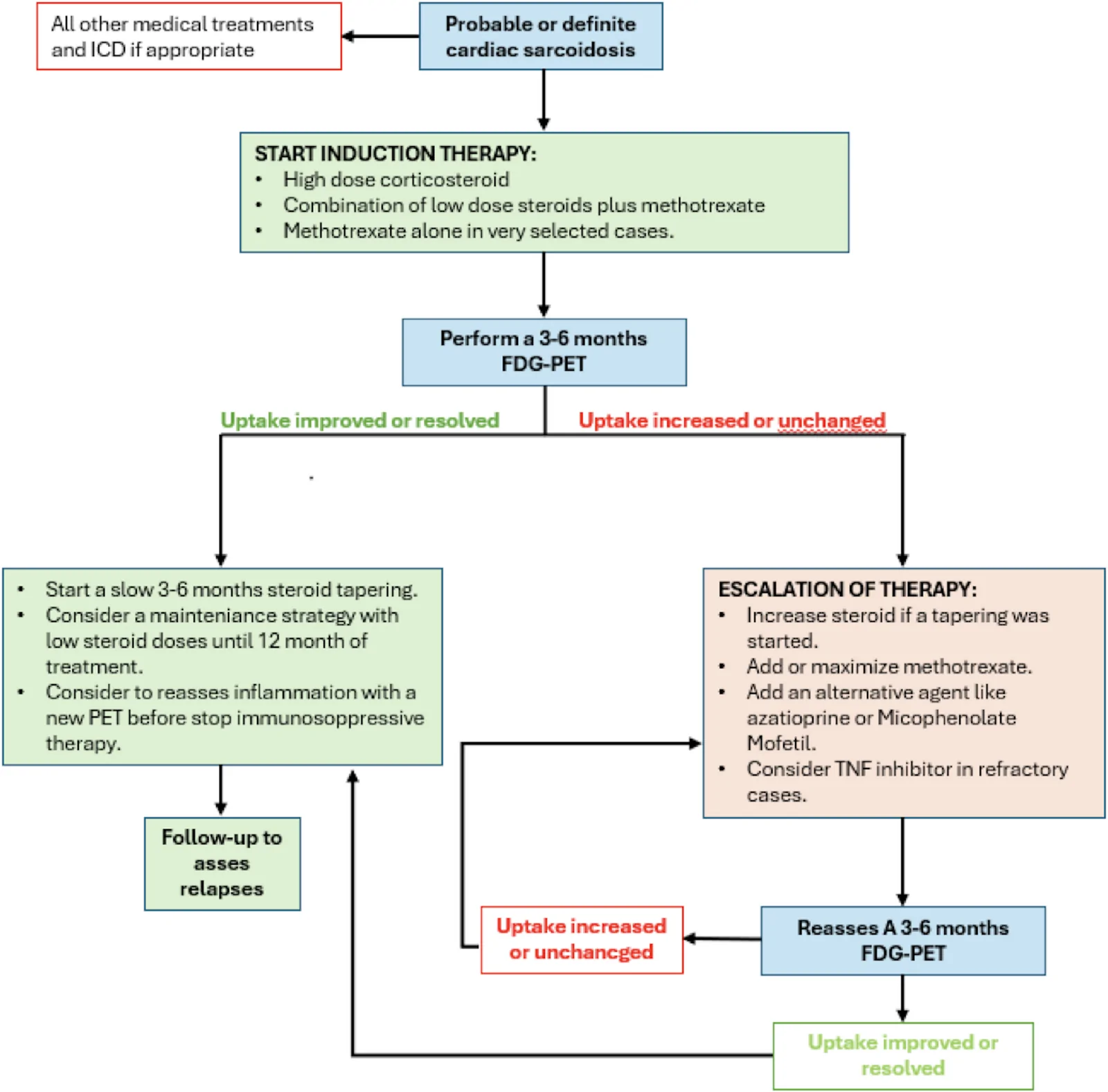
Suggested treatment algorithm for CS-related myocardial inflammation. Abbreviations: CS, cardiac sarcoidosis; FDG-PET, ^18F-fluorodeoxyglucose positron emission tomography; ICD, implantable cardioverter-defibrillator; PET, positron emission tomography; TNF, tumour necrosis factor

**Fig. 4:**
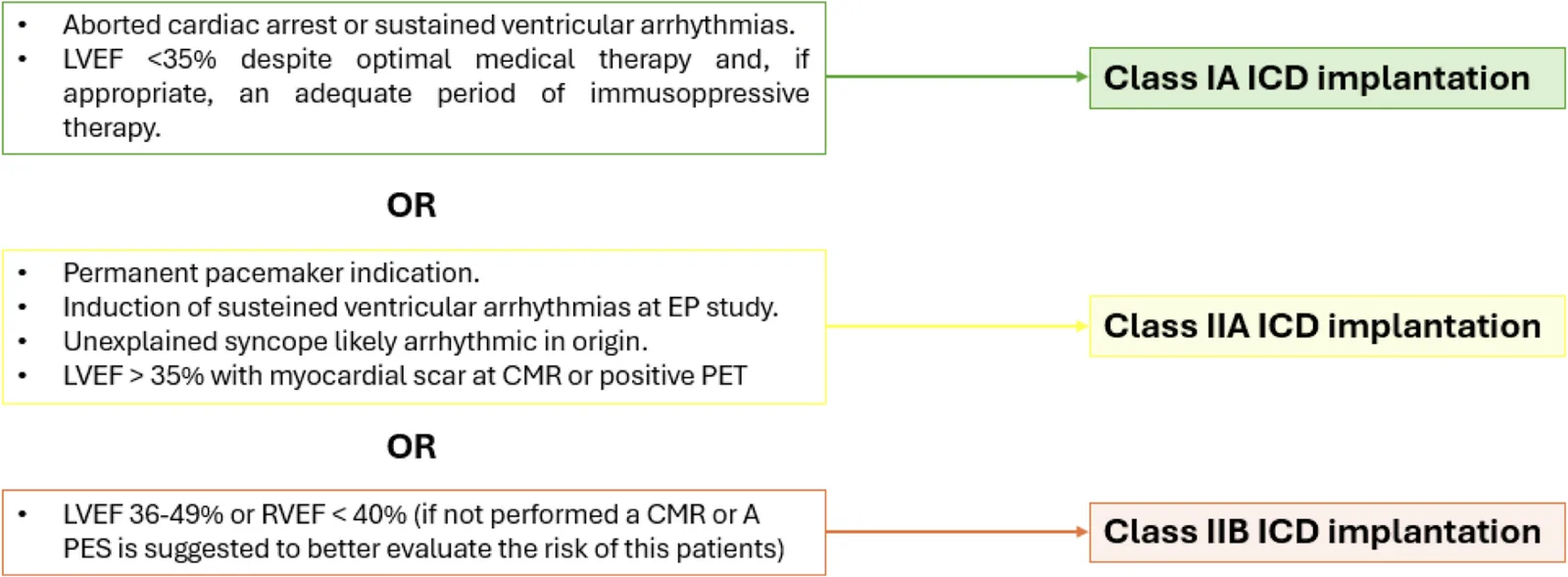
ICD indications in cardiac sarcoidosis. Abbreviations: CMR, cardiac magnetic resonance; EP, electrophysiological; ICD, implantable cardioverter-defibrillator; LVEF, left ventricular ejection fraction; PES, programmed electrical stimulation; PET, positron emission tomography; RVEF, right ventricular ejection fraction

A proposed treatment algorithm is summarized in Fig. 3, while ICD indications in CS are summarized in Fig. 4. High-risk features (like high-grade AV block, symptomatic VT, or reduced LVEF) prompt immediate immunosuppression and consideration of ICD, whereas lower-risk cases might be monitored more closely with serial imaging before initiating therapy. FDG-PET is often used to tailor therapy duration: after 3–6 months of immunosuppression, a repeat PET is done – if inflammation has resolved, a gradual taper is pursued; if it persists, therapy is intensified (adding or increasing second-line agents or trying biologics).

### Prognosis and Outcomes

Cardiac sarcoidosis (CS) is a potentially life-threatening condition that, if left untreated, can progress and lead to fatal outcomes. However, early diagnosis and advancements in therapeutic strategies have significantly improved patient prognoses. Historical data from the 1990s indicated poor survival rates, with 1-year survival around 77% and 5-year survival approximately 60% without therapy. In contrast, contemporary studies report 5-year survival rates between 90% and 96%, and 10-year survival rates ranging from 80% to 90%, aligning CS outcomes more closely with other non-ischemic cardiomyopathies rather than rapidly fatal conditions [43]. CS is characterized by a relapsing-remitting course. Even after achieving apparent remission, patients may experience relapses manifested by new arrhythmias or areas of active inflammation. Close monitoring is essential, particularly during and after corticosteroid tapering. Studies indicate that approximately 50% of patients in remission experience relapse, with higher risk associated with greater late gadolinium enhancement (LGE) on cardiac magnetic resonance imaging and isolated CS [44]. Consequently, many clinicians advocate for prolonged low-dose maintenance therapy in patients with life-threatening initial presentations, although definitive endpoints for therapy cessation remain undefined.

A subset of CS patients progresses to advanced heart failure (HF) despite maximal medical therapy, accounting for approximately 0.5% to 1% of heart transplants in certain regions. Outcomes post-transplantation are generally favorable, with 1-, 5-, and 10-year survival rates approximating 90%, 77%, and 60%, respectively, comparable to those in patients with non-sarcoid cardiomyopathies [45]. Recurrence of sarcoid granulomas in the transplanted heart is infrequent, estimated between 5% and 17%, and often clinically mild. If recurrence occurs, it is typically manageable with augmented immunosuppression. Therefore, heart transplantation remains a viable and life-saving option for end-stage CS and should not be precluded based on concerns of recurrences [46].

### Follow-Up and Monitoring

There is no universally accepted surveillance protocol for cardiac sarcoidosis (CS); however, follow-up strategies are typically individualized based on clinical status and initial disease severity. Key components of routine monitoring include:

- **Clinical assessment**, including evaluation of symptoms.
- **Electrocardiogram (ECG)** and **rhythm monitoring** to detect arrhythmias.
- **Echocardiography** to assess cardiac function.

These evaluations are generally recommended at intervals of every 6 to 12 months.

The optimal frequency of advanced imaging modalities, such as 18 F-fluorodeoxyglucose positron emission tomography (FDG-PET), has not been definitively established. Nonetheless, many experts advocate for a follow-up FDG-PET scan approximately 6 months after initiating or modifying immunosuppressive therapy to assess for residual active inflammation. If the scan shows no abnormal uptake, clinicians may consider tapering corticosteroids while continuing clinical monitoring. Conversely, if the scan remains positive, it may be appropriate to maintain or intensify therapy and repeat imaging in another 6 to 12 months. Once sustained remission is achieved, routine FDG-PET imaging may be deferred, reserving its use for instances of symptom recurrence [47].

Some clinicians employ a multimodal imaging approach, alternating between FDG-PET and cardiac magnetic resonance imaging (CMR) at intervals (e.g., FDG-PET at 6 months, CMR at 12–18 months) to obtain complementary information on inflammation and fibrosis [48].

Additionally, coordination with other specialists is crucial for comprehensive systemic disease monitoring. For instance, annual ophthalmologic examinations may be indicated for ocular sarcoidosis, and pulmonary function tests or imaging studies may be necessary for pulmonary involvement, depending on the organs affected.

## Knowledge Gaps and Future Directions

Despite major advances in the understanding and treatment of cardiac sarcoidosis (CS), critical knowledge gaps persist. Ongoing and future research aims to address several key areas to enhance diagnosis, management, and patient outcomes:

1. **Randomized Therapy Trials.** Currently, there are no randomized controlled trials (RCTs) that definitively establish the efficacy of corticosteroids versus no treatment in CS, nor the optimal dosing strategy. Most treatment regimens are based on observational studies or systemic sarcoidosis data. The CHASM-CS-RCT trial (Phase III) is underway to compare high-dose prednisone with a lower-dose prednisone plus methotrexate (MTX) regimen. This trial may clarify whether a steroid-sparing approach can provide similar benefits with fewer side effects. Results expected in the next 1–2 years could redefine first-line therapy recommendations.
2. **Advancements in Imaging.** New positron emission tomography (PET) tracers, such as somatostatin receptor-based agents (e.g., ^68Ga-DOTATATE), are under investigation for improved specificity in detecting granulomatous inflammation. Simultaneous PET/cardiac MRI (CMR) imaging enables accurate correlation of inflammation with scar tissue. Quantitative imaging metrics—including LGE volume, standardized uptake values (SUVs) on PET, and T1 mapping on CMR—may offer better risk stratification and response monitoring than current qualitative assessments. Automated image analysis and machine learning tools are also being explored to improve predictive accuracy.
3. **Biomarkers for Disease Activity**. A reliable serum biomarker for tracking disease activity in CS would be a transformative tool. Research into microRNA profiles and other “omics” approaches is ongoing to identify unique molecular signatures of CS. While no biomarker has yet matched the sensitivity and specificity of imaging, future combinations may help predict disease activity and outcomes more effectively.
4. **Genetics and Molecular Mechanisms.** Although some HLA associations have been identified, the genetic factors driving cardiac involvement in sarcoidosis remain unclear. Molecular studies of granulomas could uncover new therapeutic targets. For example, the role of macrophage polarization—from inflammatory M1 to fibrotic M2 types—may offer insight into the transition from inflammation to fibrosis and scarring.
5. **Device Therapy and Risk Stratification.** Current guidelines support broad use of implantable cardioverter-defibrillators (ICDs), but this may lead to overtreatment. Improved tools for risk stratification are needed to identify which patients—especially those with moderate ejection fraction (EF) or limited late gadolinium enhancement (LGE)—will genuinely benefit from ICDs versus those who may be monitored safely.
6. **Advances in Electrophysiology.** Catheter ablation for ventricular tachycardia (VT) in CS continues to evolve. Emerging technologies, including high-definition electroanatomic mapping and epicardial access techniques, may improve outcomes, particularly in patients with scar tissue on the heart’s outer surface.
7. **Managing Borderline or Asymptomatic Cases.** Improved imaging sensitivity has led to the detection of mild or incidental cardiac involvement in patients with systemic sarcoidosis. The clinical significance of findings like isolated LGE in asymptomatic individuals is unclear. Research is needed to determine whether early treatment alters disease progression or whether observation is safe in such cases.
8. **Big Data and Global Registries.** Due to the rarity of CS, large multicenter registries are essential for advancing knowledge. Initiatives such as the World Association of Sarcoidosis and Other Granulomatous Disorders (WASOG) registry aim to support real-world studies on prognostic indicators, treatment outcomes, and disease progression.

## Conclusions

Cardiac sarcoidosis (CS) is a complex and demanding condition. Over the past decade, increased clinical awareness and advancements in diagnostic technologies have enabled earlier and more accurate detection. Although diagnosis still often relies on clinical judgment in conjunction with guideline-based criteria, modern imaging tools now allow for the identification of cardiac involvement even without histologic confirmation in many cases.

Effective management of CS requires a comprehensive and individualized approach. High-dose corticosteroids remain the cornerstone of treatment to suppress myocardial inflammation, typically accompanied by steroid-sparing immunosuppressive agents to maintain long-term remission and minimize side effects. At the same time, proactive prevention of sudden cardiac death (SCD)—primarily through implantable cardioverter-defibrillators (ICDs)—is essential, given the unpredictable risk of life-threatening arrhythmias. ICD decision-making must go beyond left ventricular ejection fraction (LVEF) alone, incorporating factors such as scar burden, arrhythmic history, and other clinical risk indicators.

Patient outcomes have improved significantly. Once considered a highly fatal disease, CS is now increasingly managed as a chronic condition, with many patients achieving long-term survival. However, due to its relapsing-remitting nature, continuous monitoring remains essential. Longitudinal follow-up allows clinicians to detect disease recurrence, adjust therapy, and prevent complications. Multidisciplinary care—engaging cardiology, electrophysiology, pulmonology, and other specialties—is vital for managing the systemic manifestations of sarcoidosis and optimizing overall outcomes.

Nonetheless, several challenges remain. There is no universally accepted immunosuppressive regimen, and diagnostic criteria continue to evolve, particularly for isolated CS without extracardiac involvement. Risk stratification for SCD remains imperfect, often leading to empirical ICD implantation that may not always be necessary. Ongoing and future research is expected to deliver more accurate diagnostic biomarkers and targeted therapies capable of modulating the granulomatous inflammatory process with greater precision and fewer side effects than current broad-spectrum immunosuppression.

## Key References

- Cheng RK, Kittleson MM, Beavers CJ, Birnie DH, Blankstein R, Bravo PE, et al. Diagnosis and Management of Cardiac Sarcoidosis: A Scientific Statement From the American Heart Association. Circulation. 2024; 149: e1197-e1216.
  - ○ This contemporary American Heart Association scientific statement provides an authoritative framework for the diagnosis, risk stratification, and management of cardiac sarcoidosis, integrating multimodality imaging, immunosuppression, and prevention of sudden cardiac death.
- Sharma R, Kouranos V, Cooper LT, Metra M, Ristic A, Heidecker B, et al. Management of cardiac sarcoidosis. Eur Heart J. 2024; 45(30): 2697-2726.
  - ○ This comprehensive state-of-the-art review summarizes recent evidence on the diagnosis and treatment of cardiac sarcoidosis and provides a clinically useful approach to immunosuppressive therapy, arrhythmia management, heart failure, and device therapy.
- Mälkönen H, Lehtonen J, Pöyhönen P, Uusitalo V, Mäyränpää MI, Kupari M. Endomyocardial biopsy in the diagnosis of cardiac sarcoidosis. Eur J Heart Fail. 2025; 27(3): 488-497.
  - ○ This recent study re-evaluates the diagnostic value of endomyocardial biopsy in cardiac sarcoidosis and helps define clinical and imaging features associated with improved biopsy yield, supporting a more targeted approach to tissue diagnosis.

## Data Availability

No datasets were generated or analysed during the current study.

## Declarations

### Use of Artificial Intelligence

Artificial intelligence (AI) tools were used to assist with language editing, preparation of revisions, and organization of references and tabular content. No original data were analyzed by AI. All scientific statements, citations, and tables were independently verified, critically revised, and approved by the authors, who take full responsibility for the final manuscript.
